# Isolation and Characterization of 89K Pathogenicity Island-Positive ST-7 Strains of *Streptococcus suis* Serotype 2 from Healthy Pigs, Northeast China

**DOI:** 10.1100/2012/302386

**Published:** 2012-06-04

**Authors:** Shujie Wang, Peng Liu, Chunyu Li, Yafang Tan, Xuehui Cai, Dongsheng Zhou, Yongqiang Jiang

**Affiliations:** ^1^National Key Laboratory of Veterinary Biotechnology, Harbin Veterinary Research Institute, Chinese Academy of Agricultural Science, Harbin 150001, China; ^2^State Key Laboratory of Pathogen and Biosecurity, Beijing Institute of Microbiology and Epidemiology, Beijing 100071, China

## Abstract

*Streptococcus suis* is a swine pathogen which can also cause severe infection, such as meningitis, and streptococcal-like toxic shock syndrome (STSS), in humans. In China, most of the *S. suis* infections in humans were reported in the southern areas with warm and humid climates, but little attention had been paid to the northern areas. Data presented here showed that the virulent serotypes 1, 2, 7, and 9 of *S. suis* could be steadily isolated from the healthy pigs in the pig farms in all the three provinces of Northeast China. Notably, a majority of the serotype 2 isolates belonged to the 89K pathogenicity island-positive ST-7 clone that had historically caused the human STSS outbreaks in the Sichuan and Jiangsu provinces of China, although the human STSS case caused by *S. suis* had never been reported in northern areas of China. Data presented here indicated that the survey of *S. suis* should be expanded to or reinforced in the northern areas of China.

## 1. Introduction

*Streptococcus suis *is an important pathogen that can cause the severe systemic infection in the pigs reported worldwide [1]. *S. suis *can also be frequently isolated from other animals such as cats, dogs, and horses, and thus it is believed to be a commensal in the animal intestinal flora [2]. A total of 35 serotypes have been characterized for the *S. suis* isolates from the healthy pigs, but only a limited portion (serotypes 1 to 9, and 14) of them are responsible for the infections in pigs.

 Being a causative agent of a zoonotic disease, *S. suis* can be transmitted from pigs to humans. Since the first human case caused by* S. suis* was reported in Denmark, increasing numbers of human cases have been reported in many countries especially including South Asia [3]. Human infections generally can be manifested as meningitis, septicaemia, endocarditis, and deafness. Nearly all the human cases characterized can be ascribed to the handling/consumption of unprocessed pork meat, or to the close contact with pigs [4]. Therefore, most of the infected people are pig farmers, abattoir workers, meat inspectors, butchers, or veterinarian practitioners.

*S. suis* serotype 2 is the most highly pathogenic one of the 35 serotypes for both pigs and humans [5], and it has caused the two recent outbreaks of human infection in China, which are characterized by a streptococcal toxic shock-like syndrome (STSS), with higher-than-usual human morbidity and mortality [6]. The STSS-causing *S. suis *serotype 2 strains have acquired a 89K pathogenicity island (89KPaI) harboring multiple virulence determinants [6–8]. The acquisition of 89KPaI through gene horizontal transfer plays important roles in the rapid adaptation and increased virulence of *S. suis *serotype 2 [6–8].

In the present work, a total of 155 *S. suis* strains were isolated from the 2204 nose swabs collected from the healthy pigs distributed in all three provinces in Northeast China. At least four virulent serotypes, 1, 2, 7, and 9 were discriminated from these strains. Notably, a collection of 89KPaI-positive ST-7 strains of serotype 2 were identified for the first time in northern areas of China.

## 2. Material and Methods

### 2.1. Specimens Collection

A total of 2204 nose swabs were collected with the aseptic procedures from 2204 different healthy pigs from March to November, 2007. The pigs included growing, nursery, and finishing ones, and sows from 23 pig farms in Northeast China (Heilongjiang, Liaoning and Jilin provinces).

### 2.2. Isolation of Bacterial Strains and Genomic DNA

The selective Todd-Hewitt broth containing polymyxin B (10 mg/mL), nalidixic acid (15 mg/mL), and crystal violet (0.1 mg/mL) was used for the primary isolation of *S. suis* from the nose swab specimens [9]. Bacteria were cultured overnight on the Columbia sheep agar plates at 37°C for isolation of the genomic DNA by using a genomic DNA isolation kit (Tigan, Beijing, China).

### 2.3. PCR Detection

*S. suis* was identified by detecting a specific *gdh*-amplification product through PCR [10]. The serotypes 1, 2, 7, and 9 were identified by PCR with the previously characterized serotype-specific primers [11, 12]. PCR was also done to detect the virulence genes [13, 14] encoding muramidase-released protein (MRP), extracellular protein factor (EF), and suilysin (SLY) and to detect the SSU05_0943 gene in the 89KPaI [7].

### 2.4. Multilocus Sequence Typing Analysis (MLST)

 A previously described MLST scheme [15] was applied to the indicated *S. suis *strains. This MLST scheme involved seven housing-keeping gene loci, that is, *cpn60*, *dpr*, *recA*, *aroA*, *thrA*, *gki*, and *mutS*, which encoded 60-kDa chaperonin, putative peroxide resistance protein, homologous recombination factor A, 5-enolpyruvylshikimate 3-phosphate synthase, aspartokinase/homoserine dehydrogenase, glucose kinase, and DNA mismatch repair enzyme, respectively. PCR products were purified by using the QIAquick PCR product purification columns and then sequenced from both ends with an ABI Prism 3700 DNA analyzer system. The obtained sequences were compared with the previously defined allelic sequences in the *S. suis* MLST database (http://ssuis.mlst.net/), to identify the allelic profile or sequence type (ST) of each isolate tested herein.

### 2.5. Microarray-Based Comparative Genomic Hybridization (M-CGH)

Gene contents were compared between each paired test and reference (05ZYH33) DNAs using a whole-genome DNA microarray [16, 17] imprinted with 98% of the 2194 annotated ORFs of the *S. suis* serotype 2 strain 05ZYH33 (each ORF was printed in duplicate on a single glass slide). Each paired test and reference (05ZYH33) DNAs were labeled with difference fluorescent dyes (Cy3 or Cy5 dye) and then cohybridized to a microarray slide. Experiments were repeated in duplicate (two biological DNA probes replicates, and accordingly two microarray slides for each strain), for which the incorporated dye was reversed. The hybridized slides were scanned by using a GenePix Personal 4100A Microarray Scanner (Axon Instruments). The scanning images were processed and the data were further analyzed by using GenePix Pro 5.0 software (Axon Instruments) combined with Microsoft Excel software. Spots with signal intensity (median) in the channel of Reference DNA less than two folds of local background intensity (median) were rejected from further analysis. Spots with bad data because of slide abnormalities were discarded as well. Data normalization was performed on the remaining spots by total intensity normalization methods. A ratio of intensity (test DNA normalized intensity/reference DNA normalized intensity) was recorded for each spot and then was converted to log_2_. Genes with fewer than three data points were considered unreliable and were accordingly removed. The averaged log_2_ ratio for each remaining gene on the two replicate slides was ultimately calculated. If 20% of the strains had a gene with missing data, the gene was removed. A log⁡_2_⁡ value equal to or lower than −1 was taken as defining the absence of a gene in given strain. The binary dataset of absent (0) or present (1) sign genes among strains was displayed by the TreeView software vesion1.60 [18]. 

## 3. Results and Discussion

### 3.1. Identification and Characterization of Total *S. suis* Strains

**Figure 1:**
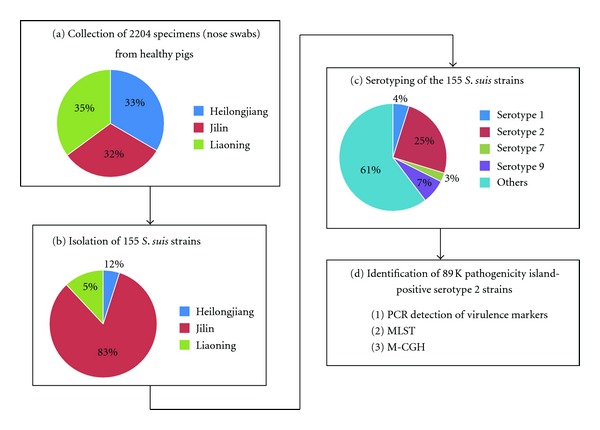
Flow charts of the analyses in this study.

The Northeast China region consists of three provinces Jilin, Heilongjiang, and Liaoning. As shown in Figure 1(a), the 2204 nose swab specimens from 2204 different healthy pigs tested could approximately equally assigned into these three provinces. From the 2204 specimens, a total of 155* S. suis* strains were isolated (Figure 1(b)), and these isolates were identified as *S. suis* by the positive PCR detection of the* S. suis*-specific *gdh* gene [10]. The three provinces Jilin, Heilongjiang, and Liaoning accounted for 83%, 12%, and 5% of the strains isolated, respectively. Accordingly, the *S. suis* isolation rate (no. of *S. suis* strain isolated/no. of specimens tested) for Jilin, Heilongjiang, and Liaoning were 18.43%, 0.95%, and 2.64%, respectively. The much higher isolation rate for Jilin might be due to the fact that the specimens from Liaoning and Heilongjiang were collected during colder months (in March, April, and October) whereas those from Jilin during warmer months (in June and July).

The serotypes 1, 2, 7, and 9 were screened by PCR for these 155 *S. suis* isolates with the previously characterized serotype-specific primers of *S. suis* [11, 12]. Accordingly, the 155 isolates were composed of 39 (25%) serotype-2 strains, 11 (7%) serotype-9 ones, 7 (4%) serotype-1 ones, 4 (3%) serotype-7 ones, and 94 (61%) ones of unknown serotypes (Figure 1(c)). The PCR assays showed that all the 39 serotype 2 strains harbored the three virulence genes encoding MRP, EF, and SLY. These results indicated the highly virulent serotype 2 strains could be frequently isolated from the healthy pigs in Northeast China. 

### 3.2. Identification and Characterization of 89KPaI-Positive *S. suis *Serotype 2

The SSU05_0943 gene in the 89KPaI was chosen for the PCR-based screening for this island in the 39 serotype 2 strains; 32 (82%) of these 39 strains gave positive PCR reaction, indicating that the corresponding strains potentially harbored this genomic island.

Of the above 32 strains potentially harboring the 89KPaI, 15 were arbitrarily selected for the MLST assay. All these 15 strains were identified as ST-7 with an allelic profile 1,1,1,1,1,1,3, which was the same as that of the reference strain 05ZYH33 (an 89KPaI-positive strain, with the determined genome sequence, isolated from the human STSS case [7]). ST-7, represented by the STSS-causing, 89KPaI-positive strains of *S. suis *serotype 2, emerged first in Hong Kong in 1996, and caused 28 cases of human* S. suis* infection in Jiangsu Province, China, in 1998, and another large outbreak of human infection in Sichuan Province, China, in 2005 [6–8, 19]. As a member of the ST-1 (allelic profile 1,1,1,1,1,1,1) complex, ST-7 is a single-locus variant of ST-1 with increased virulence [19], as demonstrated by the fact that the toxicity of ST-7 to peripheral blood mononuclear cells is greater than that of ST-1 [19].

**Figure 2:**
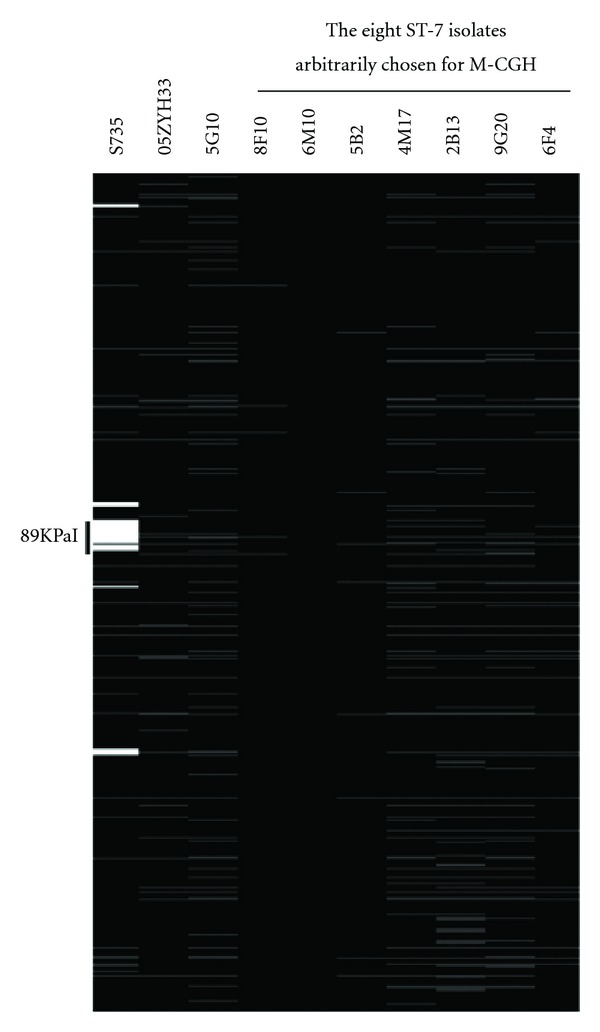
Schematic presentation of M-CGH data. Each column represented a strain, while each row standed for a different gene. The strain names were presented on the top. Genes were arranged according to the genomic location of the strain 05ZYH33. For each individual strain, the presence of a gene was represented by a black box, whereas the absence of a gene corresponded to a white box, and the grey area indicated the missing data.

Of the above 15 ST-7 strains determined by MLST, eight were arbitrarily chosen for the further M-CGH analysis (Figure 2). M-CGH has been established as a standard method for the bacterial comparative genome analysis in our laboratory [20, 21]. In the present work, a total of *S. suis* 1918 genes were included in the final microarray dataset, and each gene was categorized as either present (1), absent (0), or missing data for each strain. The eight ST-7 strains tested harbored all the 89KPaI genes imprinted on the microarray, and they gave the gene profiles almost the same as that of the reference 89KPaI-positive ST-7 strain 05ZYH33, indicating the high clonal genomic content of 05ZYH33 and the above eight ST-7 strains. Included also in the M-CGH analysis was S735 [22] that was a ST-1 strain (MRP+, EF+, SLY+, and 89KPaI-; serotype 2) isolated from a diseased pig in Netherlands in 1963. For S735, the absence of various genome loci (including 89KPaI) of 05ZYH33 was detected by M-CGH. It could be solidly concluded that the above eight strains, characterized by both MLST and M-CGH, belonged to the epidemic 89KPaI-positive ST-7 clone of *S. suis *serotype 2 that has historically cause the human STSS outbreaks in China.

### 3.3. Concluding Remarks

In China, most of the *S. suis* infections in humans were reported in the southern areas (such as Sichuan, Jiangsu, and Guangdong provinces, and Hongkong) with higher environmental humidity and temperature [23]. However, little attention had been paid to the northern areas of China. Our results showed that the virulent serotypes 1, 2, 7, and 9 could be steadily isolated from the healthy pigs in the pig farms in Northeast China. To the best of our knowledge, this is the first report of the isolation of the 89KPaI-positive ST-7 strains of *S. suis* serotype 2 in Northeast China. Although the human STSS case caused by *S. suis* has never been reported in northern areas of China, a routine survey of *S. suis* in these geographic regions is needed.
